# ADSCs and adipocytes are the main producers in the autotaxin–lysophosphatidic acid axis of breast cancer and healthy mammary tissue in vitro

**DOI:** 10.1186/s12885-018-5166-z

**Published:** 2018-12-19

**Authors:** Rafael Schmid, Katharina Wolf, Jan W. Robering, Selina Strauß, Pamela L. Strissel, Reiner Strick, Matthias Rübner, Peter A. Fasching, Raymund E. Horch, Andreas E. Kremer, Anja M. Boos, Annika Weigand

**Affiliations:** 1 Department of Plastic and Hand Surgery and Laboratory for Tissue Engineering and Regenerative Medicine, University Hospital of Erlangen, Friedrich-Alexander-University Erlangen-Nürnberg (FAU), Krankenhausstr. 12, 91054 Erlangen, Germany; 2 Department of Medicine I, University Hospital of Erlangen, Friedrich-Alexander-University Erlangen-Nürnberg (FAU), Erlangen, Germany; 3 Laboratory for Molecular Medicine, Department of Gynecology and Obstetrics, University Hospital of Erlangen, Friedrich-Alexander-University Erlangen-Nürnberg (FAU), Erlangen, Germany; 4 Department of Gynecology and Obstetrics, Comprehensive Cancer Center Erlangen ER-EMN, University Hospital Erlangen, Friedrich-Alexander-University Erlangen-Nürnberg (FAU), Erlangen, Germany

**Keywords:** Breast cancer, Autotaxin, Lysophosphatidic acid, Therapy, ADSC

## Abstract

**Background:**

Breast cancer is the most common malignancy in women affecting one out of eight females throughout their lives. Autotaxin (ATX) is upregulated in breast cancer which results in increased lysophosphatidic acid (LPA) formation within the tumor. This study’s aim was to identify the role of different mammary cell populations within the ATX–LPA axis.

**Methods:**

Epithelial-cell-adhesion-molecule-positive (EpCAM) and -negative cells from breast tumors, adipose-derived stem cells (ADSCs) of tumor-adjacent and tumor-distant mammary fat were isolated and compared to healthy ADSCs, mammary epithelial cells (HMECs), and mesenchymal cells (MES) of healthy mammary tissue (*n* = 4 each) and further to well-established breast (cancer) cell lines.

**Results:**

mRNA expression analyses revealed that ADSCs and MES largely expressed LPA receptor 1 (*LPAR1*) while epithelial cells mainly expressed *LPAR6*. LPA 18:1 activated all the cell populations and cell lines by rise in cytosolic free calcium concentrations. MES and ADSCs expressed ATX whereas epithelial cells did not. ADSCs revealed the highest expression in ATX with a significant decline after adipogenic differentiation in healthy ADSCs, whereas ATX expression increased in ADSCs from tumor patients. Breast (cancer) cell lines did not express ATX. Transmigration of MES was stimulated by LPA whereas an inhibitory effect was observed in epithelial cells with no differences between tumors and healthy cells. Triple-negative breast cancer (TNBC) cell lines were also stimulated and the transmigration partly inhibited using the LPA receptor antagonist Ki16425.

**Conclusions:**

We here show that each mammary cell population plays a different role in the ATX–LPA axis with ADSCs and adipocytes being the main source of ATX in tumor patients in our experimental setting. Inhibitors of this axis may therefore present a valuable target for pharmacological therapies.

## Background

As breast cancer affects one out of eight women throughout their lives, it is the most common malignancy amongst women. Nevertheless, due to improved cancer detection and treatment, mortality has decreased over the past 20 years [1], however, treatments often fail. Metastatic breast tumors tend to develop resistance to treatments with chemotherapeutics or radiotherapy. Current targeted therapies seem to offer significant improvement in some but not all of these cases [2]. Another candidate for target therapies could be autotaxin (ATX), a gene that is among the top 40 most up-regulated genes in metastatic cancer cell lines [3].

ATX was originally identified as autocrine cell motility factor in conditioned medium of melanoma cells [4]. Initially, it was believed to function mainly as pyrophosphatase and was therefore named ectonucleotide pyrophosphatase/phosphodiesterase 2, ENPP2 [5]. Later, its lysophospholipase activity was recognized which is responsible for hydrolyzing lysophosphatidyl choline (LPC) into lysophosphatidic acid (LPA) [6, 7]. LPA is a small bioactive phospholipid which signals via at least six different G-protein coupled receptors entitled LPAR1–6 [8–10]. The LPA–ATX axis influences several cellular pathways [11] including those concerning cell proliferation and migration especially of tumor cells [12–16]. In recent studies, the stimulatory influence of adipocyte derived ATX [17, 18] on breast cancer cell proliferation and breast cancer progression was revealed [19–21].

ATX expression and thereby LPA synthesis is upregulated in invasive breast cancers [22]. Benesch et al. [19] published that the combination of a breast tumor and the surrounding fat tissue leads to a vicious cycle. Increased inflammatory cytokines lead to increased ATX expression and augmented ATX levels within the tumor. The tumor surrounding fat tissue of the breast represents a major source of ATX which is in contrast to other carcinomas such as thyroid cancer [23]. Elevated ATX and LPA levels reduce the capacity of inducing apoptosis in tumor cells, thereby contributing to the resistance to chemotherapeutics and radiotherapy [24]. A recent study of Meng et al. [20] indicated that radiotherapy further increased ATX production in adipose tissue. Further, high levels of the LPAR3 in epithelial cells are also associated with a more aggressive tumor progression [25]. Dysfunctional regulation of the ATX–LPA axis, even on the receptor site, could promote the formation of breast tumors. In transgenic mice with overexpression of ATX, LPAR1, LPAR2, or LPAR3, spontaneous mammary tumor formation has been reported [26].

Many findings on ATX in breast cancer are based on tumor cell line models and so it remains unclear which cell populations within the tumor environment secrete ATX and thereby contribute to the vicious cycle of tumor progression [19]. We here evaluate this model with different primary human mammary-derived cell types of breast cancer patients and compare these results to primary mammary cells of healthy tissue and several breast (cancer) cell lines. This study can help to analyze which cells are the source of elevated ATX levels in breast cancer and clarify tumorous cell–cell interactions as well as it may offer an opportunity for targeted therapies.

## Methods

### Isolation of epithelial and mesenchymal tumor cells

For the isolation of epithelial and mesenchymal cells from luminal B or triple-negative mammary carcinomas (TNBC) (*n* = 4 in total), a human Tumor Dissociation Kit (Miltenyi Biotec GmbH, Bergisch Gladbach, Germany) was used.

The carcinoma was washed ten times with PBS (Sigma-Aldrich, St. Louis, MO, USA). The tumor was cut into small pieces of 2–4 mm^3^. The kit’s enzymes were used according the manufacturer’s protocol in DMEM (Biochrom, Berlin, Germany) and put into a gentleMACS C Tube (Miltenyi Biotec GmbH) together with the tumor. A gentleMACS™ Octo Dissociator was used to run program 37C_h_TDK_3 followed by program m_imptumor_01 and filtration through a 70-μm MACS® SmartStrainer (all from Miltenyi Biotec GmbH) according to the manual. The resulting suspension was centrifuged at 300 g for 7 min. The pellet was resuspended in 300 μl MACS buffer (0.5% bovine serum albumin [BSA], 2 mM EDTA in PBS adjusted to pH 7.2 [Sigma-Aldrich]) and 100 μl human FcR Blocking Reagent as well as 100 μl CD326 (EpCAM) MicroBeads, human (all from Miltenyi Biotec GmbH) were added. The magnetic separation was performed in LS Columns and a MidiMACS™ Separator (Miltenyi Biotec GmbH) using the MACS buffer. This epithelial-cell-adhesion-molecule-positive (EpCAM) fraction, named epithelial tumor cells (BCC), was centrifuged at 300 g for 10 min and seeded in Mammary Epithelial Cell Medium (ScienCell Research Laboratories, Carlsbad, CA, USA) supplemented with 100 U/ml penicillin and 0.1 mg/ml streptomycin (Sigma-Aldrich) as well as 5% FBS Superior (Biochrom) onto collagen-coated (6 μg/cm^2^ rat tail collagen type I [Sigma-Aldrich]) 6-well plates. The medium was changed to medium without FBS after 24 h.

The unlabeled fraction was collected and further sorted using CD31 MicroBeads, human in an MS Column and a MiniMACS™ Separator (all from Miltenyi Biotec GmbH). The CD31-negative flow through, named mesenchymal tumor cells (MES t) was cultured in collagen-coated 25-cm^2^ tissue culture flasks using the EpiCult™-C Human Medium Kit (STEMCELL Technologies Germany GmbH, Cologne, Germany) supplemented with 2 mM L-glutamine (Sigma-Aldrich), penicillin-streptomycin (100 U/ml, 0.1 mg/ml), 5% FBS superior, and 0.48 μg/ml hydrocortisone (STEMCELL Technologies Germany GmbH). The first medium change was performed after 48 h. After one week the media for BCC and MES were replaced with media without penicillin-streptomycin.

### Isolation of healthy, tumor-adjacent, and tumor-distant adipose-derived stem cells

Adipose-derived stem cells (ADSCs) were either isolated from healthy tissue (ADSC h, *n* = 4) or from luminal B or triple-negative mammary carcinoma patients (*n* = 4 in total).

Tumor-adjacent ADSCs (ADSC ta) were isolated from fat that was directly surrounding the tumor, tumor distant ADSCs (ADSC td) were from at least 10 cm distant mammary fat tissue.

Each tissue was cut into small pieces of less than 2 mm^3^. They were incubated in 20 ml collagenase solution (0.1% collagenase I [Biochrom] in PBS) and incubated at 37 °C for 120 min on a tube roller. The digestion was stopped using 20 ml Gibco™ MEM α including GlutaMAX™ Supplement (Thermo Fisher Scientific Inc., Waltham, MA, USA) and 10% FBS Superior. This was followed by a centrifugation step (400 g, 10 min). The pellet was resuspended in MEM α with 10% FBS and penicillin-streptomycin (100 U/ml, 0.1 mg/ml) and filtered through a 70-μm cell strainer. The cells were seeded into 75-cm^2^ cell culture flasks. The first medium change was done after 48 h and the antibiotics omitted after 1 week.

### Isolation of normal mammary epithelial cells (HMEC), normal mesenchymal cells (MES h) and normal adipose-derived stem cells (ADSC h)

For the isolation of normal mammary epithelial cells (HMEC), healthy mammary tissue (*n* = 4), was used, as described previously [27].

Summarized, the mammary tissue was cut into small pieces, followed by enzymatic incubation periods with collagenase, hyaluronidase, and trypsin and further cell fractionation. Epithelial cells were successfully separated with > 95% purity from mesenchymal cells using this protocol. The patient-matching ADSC h were isolated from breast fat tissue using collagenase digestion and incubation in red blood cell lysis buffer as previously described [27].

Like BCC and MES t, HMEC and MES h were cultivated in collagen-coated 75-cm^2^ culture flasks, in Mammary Epithelial Cell Medium respectively EpiCult™-C Human Medium supplemented with 5% FBS, 2 mM L-glutamine, and hydrocortisone (0.48 μg/ml). ADSC h were cultivated in MEM α supplemented with 10% FBS.

Medium was changed every 2–3 days. All cells were incubated at 37 °C and 5% CO_2_. Cells were used in passage 4–8.

### Cell culture of breast (cancer) cell lines

Different cell lines (all from American Type Culture Collection ATCC, Manassas, VA, USA) were used to compare the results of primarily isolated cells to established models. The mammary epithelial cell line MCF-10A (ATCC® CRL-10317™) was cultivated in Mammary Epithelial Cell Growth Medium (PromoCell GmbH, Heidelberg, Germany) with the addition of 100 ng/ml cholera toxin (Sigma Aldrich). Furthermore, we used six different breast cancer cell lines. BT-474 (ATCC® HTB-20™, luminal B) were cultivated in Hybri-Care Medium (ATCC), 10% FBS, 1.5 g/l NaHCO_3_ (Sigma Aldrich). MCF-7 (ATCC® HTB-22™, HR-positive) were cultivated in DMEM, 10% FBS, 2 mM L-glutamine. MDA-MB-231 (ATCC® HTB-26™, TNBC, mesenchymal-like) were cultivated in DMEM, 10% FBS, 2 mM L-glutamine. SKBR3 (ATCC® HTB-30™, HER2-positive) were cultivated in McCoy’s 5a Modified Medium (Thermo Fisher Scientific Inc.), 10% FBS. MDA-MB-468 (ATCC® HTB-132™, TNBC, basal-like) were cultivated in DMEM/F12 Medium (Biochrom), 10% FBS. T-47D (ATCC® HTB-133™, Luminal A) were cultivated in RPMI-1640 (Thermo Fisher Scientific Inc.), 10% FBS.

### Preparation of cell culture supernatants and cell pellets for ELISA and qPCR

The different cells were seeded at a density of 40,000 cells per 12-well into four wells. For BCC, HMEC and MES, collagen-coating was performed in advance. At a confluency of 95%, two wells were used for RNA isolation and to prepare the supernatants as conditioned media (CM).

For later RNA isolation, the wells were washed with PBS and the cells detached using Accutase® (Sigma-Aldrich, St. Louis, MO, USA) and the pellet directly frozen in liquid nitrogen. For CM, the cells were washed with PBS once and each well filled with 800 μl corresponding medium without FBS or supplements of animal origin (e.g. bovine pituitary extract). ADSCs and epithelial cells received their basal medium; MES received EpiCult™-C solely supplemented with L-glutamine and hydrocortisone. After 24 h the medium was frozen at − 80 °C for later ELISA-measurements.

Breast (cancer) cell lines were handled similarly and the CM obtained from the corresponding media without FBS.

### Adipogenic differentiation of ADSCs

Adipogenic differentiation of the ADSCs was achieved using a mesenchymal stem cell adipogenic differentiation medium (PELOBIOTECH GmbH, Planegg/Martinsried, Germany) according to the manufacturer’s protocol. ADSCs were seeded at a density of 40,000 cells per 12-well into four wells. Two wells were used for adipogenic differentiation, two as control. After reaching full confluency in the standard culture medium, the kit’s media were used for 20 days (the controls were cultivated in the standard culture medium), followed by the preparation of CM and isolation of RNA.

### RNA isolation and real-time qPCR

RNA was isolated using QIAGEN’s RNeasy Mini Kit together with the QIAshredder (QIAGEN, Hilden, Germany) according to the manufacturer’s protocols.

RNA concentration and quality was determined using a NanoDrop ND1000 Spectrophotometer (Thermo Fisher Scientific). Complementary DNA was synthesized with an oligo-dT primer and the SCRIPT cDNA Synthesis Kit (Jena Bioscience, Jena, Germany). A control sample without reverse transcriptase was carried along. Real-time PCR measurements were conducted with the SensiFAST™ SYBR® No-ROX Kit (Bioline, London, UK) in triplicates for 40 cycles (95 °C 5 s, 60 °C 10 s, 72 °C 10 s) in a CFX Connect qPCR System (Bio-Rad Laboratories, Hercules, CA, USA). Detected transcript levels were normalized to the housekeeping gene hypoxanthine-guanine phosphoribosyltransferase (*HPRT*) using the 2^-ΔCq^ method [28]. The primer sequences listed in Table 1 were used.

**Table 1:** List of primers

	Forward Primer (5′- > 3′)	Reverse Primer (5′- > 3′)
*Human HPRT*	AGTTCTGTGGCCATCTGCTT	GGCTTTGTATTTTGCTTTTCCAGT
*Human ATX*	CCTGCAGTGCTTTATCGGA	TCAGATGGTCAGGAACGCTG
*Human LPAR1*	CTCGGCATAGTTCTGGACCC	TTCTCATAGGCCAGCACGTC
*Human LPAR2*	TACCGAGAGACCACGCTCAG	GCCTAAACCATCCAGGAGCA
*Human LPAR3*	CCGCATACAAGTGGGTCCAT	GGGGTCCAGCATACCACAAA
*Human LPAR4*	AAAGATCATGTACCCAATCACCTT	CTTAAACAGGGACTCCATTCTGAT
*Human LPAR5*	CGCCATCTTCCAGATGAAC	TAGCGGTCCACGTTGATG
*Human LPAR6*	GGTAAGCGTTAACAGCTCCCACT	TTTGAGGACGCAGATGAAAATGT

### Fluorometric measurement of cytosolic free calcium levels

For the measurement of cytosolic free calcium levels, 20,000 cells were seeded into 96-well cell culture plates 2 days before measurement. Then, they were washed twice with Hank’s balanced salt solution (HBSS) containing 0.2% BSA and 10 mM HEPES (Sigma-Aldrich). Cells were incubated with 10 μM Fura-2 AM in HBSS containing BSA, HEPES and 0,25% pluronic acid (Sigma-Aldrich) for 60 min at 37 °C, were washed twice and 100 μl HBSS+HEPES+BSA were added to each well. Analyses were performed in a microplate fluorometer with integrated pipetting system (BMG Labtech NOVOstar, Offenburg, Germany) at 340 nm resp. 380 nm for excitation and 510 nm for emission. Cells were allowed to adapt to 37 °C for 10 min before LPA 18:1 (Avanti Polar Lipids Inc., Alabaster, AL, USA), solved in PBS containing 0.2% human serum albumin in indicated concentrations, was added. In cell line experiments, Ki16425 (Sigma-Aldrich) was used as LPA receptor antagonist in concentrations of 0, 2, or 20 μM 1 min prior to the stimulation with LPA. The change in cytosolic free calcium ([Ca^2+^]_i_) was calculated by dividing the measured fluorescence intensity at 510 nm with different excitation wavelengths (ratio 340 nm/380 nm). The baseline levels were subtracted. Adenosine triphosphate was used as a positive control, PBS with 0.2% human serum albumin as a negative control.

### ELISA measurements

Levels of secreted ATX in the CM were detected by a highly sensitive ELISA Kit (Human ENPP-2/Autotaxin DuoSet ELISA in combination with DuoSet ELISA Ancillary Reagent Kit 2, R&D Systems, Minneapolis, MN, USA) according to the manufacturer’s instructions using the NOVOstar.

### Transmigration assay

For the transmigration, Boyden chambers of PET-membrane transwell inserts (ThinCert™, Greiner Bio-One GmbH, Frickenhausen, Germany) with a growth surface of 33.6 mm^2^ and a pore size of 8 μm were used. The transwells were transferred into a 24-well plate and seeded with 50,000 cells in 300 μl in duplicates with reduced Mammary Epithelial Cell Medium (containing 5% of the normal supplement amount) or Epicult-C solely supplemented with L-glutamine and hydrocortisone.

The lower chamber was filled with the corresponding medium supplemented to either 1 μM or 0.1 μM LPA 18:1 dissolved in 0.2% fatty acid free BSA (Sigma) in PBS. A negative control without LPA but with BSA was also performed.

After an incubation period of 8 h at 37 °C and 5% CO_2_, the transwells were washed, fixed with ice-cold methanol (10 min) and stained with DAPI (1 μg/ml, 10 min) (Life Technologies, Carlsbad, CA, USA). The cells in the inner part of the transwells were carefully wiped with a cotton swab to remove the non-migrated cells. Cells that migrated through the membrane were counted in 4 regions of interest (ROIs) per transwell (1 image per quadrant) in 40-fold magnification (Olympus IX83, cellSens Software, Olympus Corporation, Tokio, Japan). Quantification was performed semi-automatically using Fiji Is Just ImageJ (Fiji) 1.51u [29], an extended distribution of ImageJ.

As a comparison, the transmigration assay was also performed with the breast (cancer) cell lines. Here, Ki16425 (2 μM) was used as LPA receptor antagonist in experiments with MDA-MB-231 as well as MDA-MB-468. The assay itself was performed in reduced media without FBS.

### Statistics

All the experiments were performed with *n* = 4 except for the calcium imaging of the cell lines (*n* = 3) and the ATX ELISA and the qPCR of the individual cell lines (*n* = 1). Error bars indicate SD. Statistical analysis was performed using SPSS v.21.0 software (SPSS Software/IBM, Armonk, NY, USA). Differences between groups were analyzed using the Kruskal–Wallis H test followed by a Mann–Whitney U test for post-hoc analysis; the asymptotic significance was used. Significant *p*-value was set to < 0.05 and corrected with the Bonferroni correction. All figures were created with GraphPad Prism 7.00 (GraphPad Software, La Jolla, CA, USA).

## Results

### Different mammary cells respond to stimulation with LPA 18:1 but express a different LPAR profile

We successfully isolated the distinct primary cell fractions of each patient’s mammary tissue. ADSCs were isolated from tumor-adjacent (ADSC ta) and tumor-distant (ADSC td) or healthy (ADSC h) mammary fat tissue; epithelial and mesenchymal cells were isolated from mammary glands (HMEC, MES h) or mammary tumors (BCC, MES t).

**Fig. 1:**
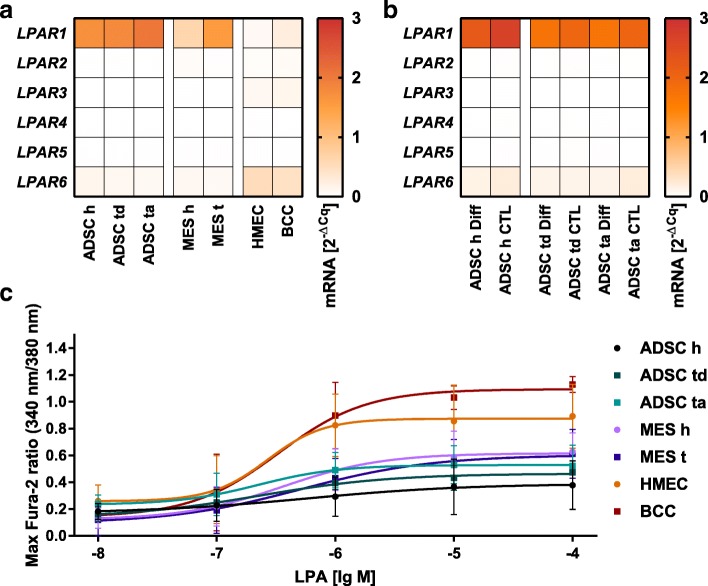
Different mammary cells respond to stimulation with LPA 18:1 but express a different LPAR profile: (**a**/**b**) Mean relative *LPAR* mRNA expression. Values are calculated using the 2^-ΔCq^ method, the reference is *HPRT*; (**a**) *LPAR* mRNA profile of ADSCs, MES and HMEC/BCC from tumors and healthy tissue; (**b**) The effect of adipogenic differentiation (Diff) on the *LPAR* mRNA profile of ADSCs compared to the control (CTL). (**c**) The effect of LPA on cytosolic free calcium levels; x-axis shows the molarity of LPA 18:1 in a common logarithmic scale, y-axis shows the maximal ratio of the emission of Fura-2 (340 nm/380 nm excitation). The mean measuring points with the SD are plotted and connected with a nonlinear fit (variable slope) using GraphPad Prism 7.00, epithelial cells have a significantly higher calcium release than ADSCs and mesenchymal cells at concentrations of 1 μM LPA and above (not plotted for clarity, *p* < 0.0167, Kruskal–Wallis H test, Mann–Whitney U test with Bonferroni correction); abbreviations: healthy h, tumor-distant td, tumor-adjacent ta, tumor t, EpCAM-positive breast cancer cells BCC; *n* = 4

In order to evaluate whether any of these cells may play a role in the ATX–LPA axis, their LPA receptor mRNA profile was analyzed and their calcium release after stimulation with LPA was measured. qPCR results (Fig. 1a) comparing the mRNA expression of *LPAR* to *HPRT* indicated that ADSCs as well as MES mainly expressed *LPAR1* and to a lesser extent *LPAR6*. Epithelial cells had a different expression profile of mainly *LPAR6* but also *LPAR1*, *LPAR2*, and to a lesser extent *LPAR3*. *LPAR4* and *LPAR5* were not expressed by any of the cells. Although there were patient-dependent variations, there was no difference between cells from tumors and healthy origin.

Simulating the role of mature adipocytes in this context, ADSCs were differentiated into the adipogenic lineage. Their *LPAR* mRNA profile did not change considerably compared to the controls (Fig. 1b) but the expression of *LPAR1* was reduced marginally. Again, there was no expression difference between cells from tumors and healthy tissue or tumor-distant and tumor-adjacent ADSCs.

Calcium imaging was used to determine the effect of LPA 18:1 on cytosolic free calcium levels. After stimulation with LPA, all different cell types released Ca^2+^ into the cytosol (Fig. 1c). A concentration of 0.01 μM LPA 18:1 was sufficient to trigger a cellular response. The cytosolic free calcium levels also increased in a dose-dependent manner with increasing LPA concentrations of up to 100 μM. Epithelial cells responded to increasing LPA concentrations with a higher calcium release than MES or ADSC. This effect was significant at concentrations of 1 μM LPA and above (*p* < 0.0167). On a logarithmic scale the response of ADSCs was almost linear whereas especially healthy mammary epithelial cells and BCC, EpCAM-positive breast cancer cells, but also MES seemed to have a more sigmoidal nonlinear regression fit (plotted using GraphPad 7.00; nonlinear fit, variable slope). There was no significant difference between cells from tumors and healthy tissue.

MCF-10A expressed mainly *LPAR6*, BT-474 and T-47D expressed little amounts of *LPAR1*, MCF-7, SKBR3, and MDA-MB-468 expressed even smaller amounts of *LPAR2*, while MDA-MB-231 expressed a little of *LPAR2* and *LPAR6* compared to *HPRT*. All breast (cancer) cell lines responded to 1 μM LPA 18:1 with increasing cytosolic free calcium levels, too (Fig. 3c). T-47D had the weakest response with no reducing effect of the LPAR antagonist Ki16425. All the other cell lines showed a reduction of the calcium response with increasing concentrations of Ki16425.

### Cell type specific differences in ATX production of mammary cells

**Fig. 2:**
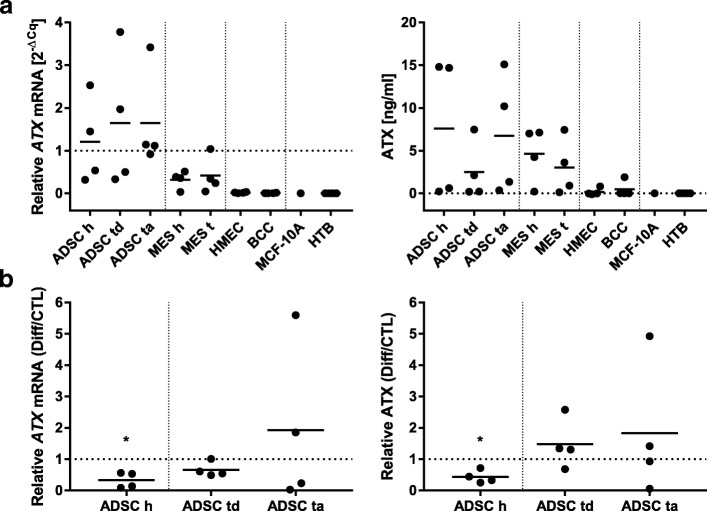
Cell type specific differences in ATX production of mammary cells: (**a**) The expression of ATX on mRNA and protein level by different mammary cells; left: relative *ATX* mRNA levels compared to *HPRT* (y-axis) of different cell types (x-axis); right: ATX protein in the conditioned media in ng/ml (y-axis) of different cell types (x-axis); ADSCs have a significantly higher ATX expression than MES on RNA level and a significantly higher expression than epithelial cells on RNA and protein level, MES also have a significantly higher ATX expression than epithelial cells both on RNA and protein level (not plotted for clarity, p < 0.0167, Kruskal–Wallis H test, Mann–Whitney U test with Bonferroni correction); (**b**) The effect of adipogenic differentiation on the ATX expression; left: relative *ATX* mRNA expression, right: relative ATX protein expression, x-axis shows the cell type, y-axis shows the ratio of differentiation (Diff) and control (CTL); the biological samples (dots) and the average (line) are plotted; abbreviations: healthy h, tumor-distant td, tumor-adjacent ta, tumor t, EpCAM-positive breast cancer cells BCC, HTB human tumor bank; *n* = 4 (MCF-10A *n* = 1, HTB *n* = 6), *p < 0.05, Kruskal–Wallis H test, Mann–Whitney U test with Bonferroni correction

An ATX-protein ELISA and qPCR for *ATX* mRNA levels were performed to analyze the expression level of ATX by different mammary cells. On average, ADSCs have a significantly higher ATX expression than MES on RNA level and a significantly higher expression than epithelial cells on RNA and protein level (*p* < 0.0167). MES also have a significantly higher ATX expression than epithelial cells both on RNA and protein level (p < 0.0167). Epithelial cells have almost no mRNA and protein expression of ATX (Fig. 2a). Again, strong differences between patients could be observed. There was no significant difference between cells from tumors and healthy tissue. Breast (cancer) cell lines did not express ATX, neither on RNA nor on protein level.

The ATX expression of ADSCs was analyzed after adipogenic differentiation (Fig. 2b) to compare them to mature adipocytes. In cells of healthy origin the adipogenic differentiation led to a significantly reduced expression of ATX on mRNA and protein level (*p* < 0.05). We observed higher variations between the tumor patients. Especially in differentiated tumor-adjacent ADSCs there was one patient with a more than fivefold expression. Compared to the controls, the average mRNA and protein ATX expression of differentiated ADSCs of tumor patients was higher than of healthy tissue.

Overall, ELISA and qPCR correlated with a Pearson correlation coefficient of 0.73.

### The transmigration of different mammary cells and breast cancer cell lines was stimulated by LPA

**Fig. 3:**
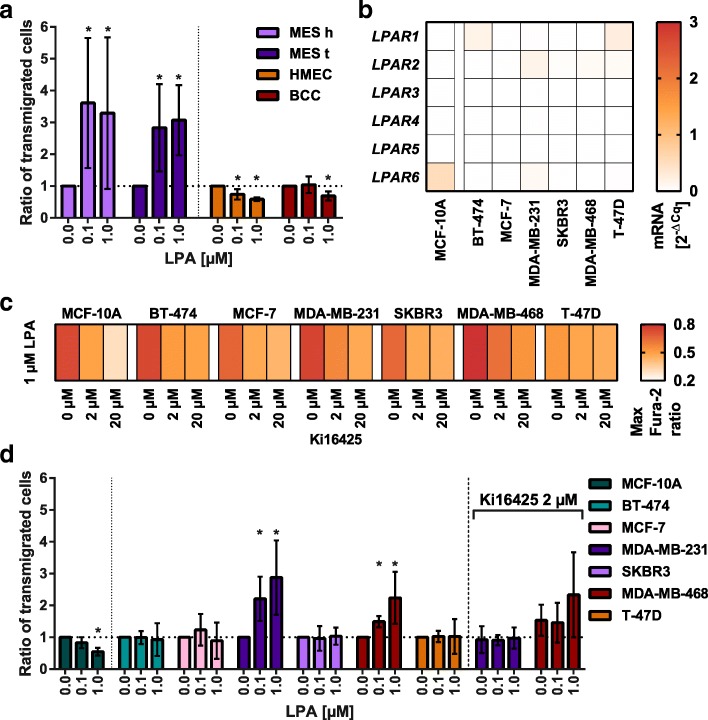
The transmigration stimulated by LPA 18:1 and profiling of breast (cancer) cell lines: (**a**) The ratio of transmigrated cells compared to the control (y-axis) in varying LPA concentrations (x-axis) of mesenchymal and epithelial cells of healthy and tumor tissue, n = 4; abbreviations: healthy h, tumor t, EpCAM-positive breast cancer cells BCC; (**b**) To *HPRT* relative *LPAR* mRNA expression of different breast (cancer) cell lines, n = 1; (**c**) Mean maximal ratio of the emission of Fura-2 (340 nm/380 nm excitation) of different breast (cancer) cell lines as effect of 1 μM LPA on cytosolic free calcium levels with the addition of the LPAR antagonist Ki16425, *n* = 3; (**d**) The ratio of transmigrated cells compared to the control (y-axis) in varying LPA concentrations (x-axis) of different breast (cancer) cell lines (n = 4) with or without the addition of 2 μM Ki16425; plotted are the means and their SD; *p < 0.0167, Kruskal–Wallis H test, Mann–Whitney U test with Bonferroni correction

A transmigration assay was performed (Fig. 3a) to evaluate the effect of LPA 18:1 on the transmigration of primary mesenchymal and epithelial mammary cells.

Both mesenchymal cells of tumors and healthy origin were significantly stimulated by 0.1 and 1 μM LPA, compared to the control without LPA (*p* < 0.0167). This resulted in approximately 200% more transmigrated cells after 8 h on average. The transmigration of epithelial cells was reduced significantly by 0.1 and 1 μM LPA, whereas the reduction was only significant at 1 μM LPA for BCC.

As a comparison, the same assay was performed with different breast (cancer) cell lines: MDA-MB-231 and MDA-MB-468 were significantly stimulated by 0.1 μM and 1 μM LPA 18:1 in their transmigration rate. With the addition of Ki16425 there was no significant stimulation compared to the control without LPA. MCF-10A had a significant inhibition in their transmigration rate at 1 μM LPA. The other breast cancer cell lines (BT-474, MCF-7, SKBR3, and T-47D) were not stimulated by LPA.

## Discussion

The role of the ATX–LPA axis in different cancers has been subject to an increasing number of publications within the recent past. Previous studies have shown that in breast cancers not the tumor but the surrounding fat tissue produces ATX [19]. To further investigate this phenomenon on a cellular level, we isolated different cell populations of breast cancer and healthy tissue and compared the results to established breast (cancer) cell lines.

Benesch et al. [19] described a vicious cycle in tumors secreting inflammatory cytokines which then stimulate fat tissue to produce ATX leading to more LPA. LPA again stimulates the tumor to proliferate and produce more cytokines. All cell types analyzed in the present investigation responded to stimulation with LPA 18:1 in the calcium imaging assay (Fig. 1c). Therefore, the vicious cycle would have had effects on all the cell populations in our setting, even though not on the same level and through different LPA receptors (Fig. 1a/b). Mesenchymal cells and ADSCs expressed mainly *LPAR1* and to some extent *LPAR6* mRNA. Epithelial cells had the highest expression of *LPAR6* and to a lesser extent *LPAR1–3* mRNA. Depending on the breast tumor subtype, elevated *LPAR3* mRNA levels have been reported. Especially HER2-positive, HR-negative, less differentiated tumors that are more aggressive are associated with increased LPAR3 levels [25]. We did not detect elevated *LPAR3* levels in our luminal B (*n* = 3) and TNBC (*n* = 1) patients. *LPAR4* and *LPAR5* were not expressed by the mammary cell populations at all. In general, a correlation of the *LPAR* mRNA profile of our cells of tumors and healthy tissue was detected. While the mammary epithelial cell line MCF-10A had a similar LPAR profile to the primarily isolated cells with a high expression of *LPAR6*, the breast cell lines revealed strikingly varying *LPAR* mRNA profiles (Fig. 3b). Compared to the housekeeping gene *HPRT*, the LPAR expression was much lower than in our primary cells. BT-474 and T-47D expressed little amounts of *LPAR1*, MCF-7, SKBR3, and MDA-MB-468 expressed even smaller amounts of *LPAR2*, while MDA-MB-231 expressed a little of *LPAR2* and *LPAR6* mRNA. This is contrary to previously published data [30] that reported MDA-MB-231 expressing higher amounts of *LPAR1* than *LPAR2* and more than 80 times higher than by MCF-10A [31]. For MCF-7, MDA-MB-468, and T-47D similar *LPAR1–3* mRNA profiles have been reported [30]. It is possible that the cell lines with differing results mutated in laboratories over time.

Interestingly, ADSCs showed an almost linear increase in cytosolic free calcium levels despite of an exponential increase in the stimulation reagent LPA 18:1 (Fig. 1c). We could see typical sigmoidal curves in our EpCAM-positive epithelial tumor cells (named BCC), in our healthy human mammary epithelial cells, and with a weaker response also in mesenchymal cells. We stimulated with a wide range of concentrations that included and exceeded the physiological plasma concentrations of LPA [32]. It is possible, that ADSCs have their plateau phase in their calcium release at even higher LPA concentrations. The breast (cancer) cell lines were also stimulated in the assay. The LPAR antagonist Ki16425 was used in increasing concentrations to evaluate possible inhibitory effects on the cell lines. In general, the cell lines showed comparable results. While 1 μM LPA stimulated the cells, increasing concentrations of Ki16425 reduced the response. Not only was the stimulation of T-47D by LPA relatively weak, also the possibility to reduce the calcium response using Ki16425 seemed to be limited. During adipogenic differentiation, we were able to detect patient-dependent differences in the amount of accumulated triglycerides and hence differences in the differentiation potential. This may also be an effect of the patient’s age [33] or physiology. While other groups reported that LPAR1 is the most abundant subtype in adipose tissue and showed a decrease of LPAR1 expression with adipogenic differentiation [34], we also found that ADSCs and adipocytes highly expressed *LPAR1*, however, we detected only a marginal reduction of *LPAR1* mRNA expression after differentiation.

We could show that especially ADSCs and not the mammary tumor cell populations expressed high levels of ATX (Fig. 2a). Epithelial cells seem to be responder in the ATX–LPA axis. In experiments with murine cell lines it was shown that differentiating preadipocytes increases the expression of ATX [18]. In contrast, undifferentiated human ADSCs produced significantly (*p* < 0.5) more ATX than differentiated adipocytes of healthy origin (Fig. 2b). In tumor patients this seemed to be reversed. In one tumor patient we even measured a higher expression of ATX after adipogenic differentiation. It is possible that this is not just a patient variance but a dysregulation as a result of the tumor’s influence and part of the tumor malignancy. Variances between different breast tumor types are also likely as e.g. also LPAR3 levels can differ significantly [25]. With no expression of ATX on RNA and protein level, the different cell lines behave like primarily isolated epithelial cells. This further supports the thesis that breast tumors do not express ATX. With a Pearson correlation coefficient of 0.73, qPCR and ELISA results of the ATX expression correlate relatively high. This indicates a good reliability of the used methods.

Concerning the effect of elevated ATX and LPA levels on higher metastasis rates, we performed transmigration assay with our cells. LPA has already been linked to increased fibroblast migration [35] as well as tumor cell migration and invasion in breast cancer cell lines [36]. Here, we evaluated the effect of LPA 18:1 on the transmigration of primary mesenchymal and epithelial mammary cells. Mesenchymal cells were stimulated in their transmigration whereas epithelial cells are inhibited. The reason can be that they signal LPA through different LPARs. Their mRNA expression profile suggests that they signal mainly via LPAR1 or LPAR6. We could not detect differences between cells isolated from tumors to healthy cells. Maybe LPA does not directly stimulate their transmigration but their proliferation or their secretion of inflammatory cytokines. Evaluating these factors should be subject of further research. When comparing the primarily isolated cells with different cell lines in their transmigration rate, it is interesting that the TNBC MDA-MB-231 and MDA-MB-468 were stimulated significantly (*p* < 0.0167) whereas the HER2-positive SKBR3, the luminal B BT-474, the luminal A MCF-7 and T-47D, and the non-cancerous MCF-10A seemed to react to LPA similar to our healthy and tumor epithelial cells with a non-stimulated or even inhibited transmigration. Our significant results showing the stimulation of MDA-MB-231 and MDA-MB-468 are also comparable to previous findings of collagen-coated transmigration assays although T-47D was not stimulated in our setting [30]. It is possible that the cells had problems in the serum-free assays, especially the long-lasting transmigration assay. The inhibitory effect of Ki16425 on MDA-MB-231 has also been subject to experiments in Matrigel-coated invasion assays [31] with similar results. With Ki16425 used as a receptor antagonist, neither MDA-MB-231 nor MDA-MB-468 were significantly stimulated by LPA 18:1 anymore. The transmigration rate of MDA-MB-231 was at the normal level with the addition of Ki16425 while it tended to be elevated for MDA-MB-468 even though not significantly.

From these results we conclude that the transmigration of tumor cells and its possibility to be stimulated by LPA 18:1 most probably depends on the tumor subtype.

Besides the effect of LPA on tumor cytokine expression or proliferation and the use of inhibitory reagents such as Ki16425, the stimulatory effect of radiotherapy on the ATX expression [20], and associated resistances to chemotherapeutics, should be studied on a cellular level. The combined knowledge will be of importance to further assess the ATX–LPA-axis as potential target for cancer therapy.

## Conclusion

The fat tissue is the main source of ATX and LPA in breast tissue. ADSCs and adipocytes contribute to the ATX expression to different extents. In healthy patients ADSCs are predominant, in tumor patients adipocytes could prevail. We demonstrated that the isolated mammary cell populations respond to LPA with a varying receptor profile. LPA stimulated the transmigration of primary mesenchymal breast cells whereas epithelial cells were inhibited without any differences between tumorous and healthy tissue. In comparison, LPA stimulated solely the two established TNBC tumor cell lines. Therefore, targeting the ATX–LPA axis may represent an additive cancer therapy for some invasive and metastatic breast tumors depending on their subtype.

## Abbreviations

ADSC: Adipose-derived stem cell
ATX: Autotaxin
BCC: EpCAM-positive cells
BSA: Bovine serum albumin
CM: Conditioned media
CTL: Control
Diff: Adipogenic differentiation
h: Healthy
HBSS: Hank’s balanced salt solution
HMEC: Human mammary epithelial cells
HPRT: Hypoxanthine-guanine phosphoribosyltransferase
HTB: Human tumor bank
LPA: Lysophosphatidic acid
LPAR: LPA receptor
LPC: Lysophosphatidylcholine
MES: Mesenchymal cells
ROI: Region of interest
ta: Tumor-adjacent
td: Tumor-distant
TNBC: Triple-negative breast cancer
